# Acknowledgment of Reviewers

**DOI:** 10.2188/jea.JE20092010AR

**Published:** 2010-11-05

The following scientsts assisted the journal by reviewing manuscripts that were submitted for consideration during the period November 1, 2008 to September 30, 2010. We gratefully acknowledge their critical evaluation and assistance in the selection of papers for publication.

**Table:** 

Adachi, Hisashi	Kurume University
Akiba, Suminori	Kagoshima Univeristy
Ando, Yuichi	The National Institute of Public Health, Japan
Arao, Takashi	Waseda University
Arima, Hisatomi	The George Institute
Arisawa, Kokichi	University of Tokushima
Asao, Keiko	University of Michigan Health System
Babazono, Akira	Kyushu University
Cattaneo, Abriano	IRCCS Burlo Garofolo
Chihara, Izumi	Jichi Medical University
Doi, Yasufumi	Kyushu University
Doi, Yuriko	National Institute of Public Health, Japan
Egawa, Ken’ichi	Meiji Yasuda Life Foundation of Health and Welfare
Fujino, Yoshihisa	University of Occupational and Environmental Health, Japan
Fujita, Toshiharu	The Institute of Statistical Mathematics
Fujiwara, Yoshinori	Tokyo Metropolitan Institute of Gerontology
Fukao, Akira	Yamagata University
Fukuda, Yoshiharu	Yamaguchi Unversity
Fukunaga, Ichiro	Institute of Health Planning
Goto, Aya	Fukushima Medical University
Haga, Hiroshi	J.F. Oberlin University
Hamajima, Nobuyuki	Nagoya University
Hamashima, Chisato	National Cancer Center
Hara, Megumi	Saga University
Hashimoto, Shuji	Fujita Health University School of Medicine
Hashizume, Masahiro	Nagasaki University
Hata, Akira	Chiba University
Hayasaka, Shinya	Hamamatsu University School of Medicine
Hayashi, Kunihiko	Gunma University
Hayashino, Yasuaki	Kyoto University
Hietasalo, Pauliina	University of Oulu
Higaki, Yasuki	Saga University
Hirokawa, Kumi	Baika Women’s University
Hirose, Kaoru	Aichi Prefectural Institute of Public Health
Hirota, Yoshio	Osaka City University
Hisashige, Akinori	Institute of HTA
Honda, Sumihisa	Nagasaki University
Honjo, Kaori	Osaka University
Honjo, Satoshi	Fukuoka East Medical Centre
Hoshuyama, Tsutomu	Ushibuka-Shimin Hospital
Hosono, Satoyo	Aichi Cancer Center
Hozawa, Atsushi	Tohoku University
Ihara, Kazushige	Tohoku University
Ikeda, Ai	Osaka University
Iki, Masayuki	Kinki University
Imai, Tomoko	Tokaigakuen University
Inaba, Yutaka	Jissen Women’s University
Inoue, Shigeru	Tokyo Medical University
Ishihara, Junko	Tokyo University of Agriculture
Ishikawa, Hirofumi	Okayama University
Ishizaki, Tatsuro	Kyoto University
Iso, Hiroyasu	Osaka University
Ito, Hidemi	Aichi Cancer Center
Iwasa, Hajime	Tokyo Metropolitan Institute of Gerontology
Iwasaki, Motoki	National Cancer Center
Kadotani, Hiroshi	Kyoto University
Kadowaki, Takashi	Shiga University of Medical Science
Kai, Ichiro	University of Tokyo
Kakehashi, Masayuki	Hiroshima University
Kamioka, Hiroharu	Physical and Health Education
Kamo, Kenichi	Sapporo Medical University
Kanda, Hideyuki	Fukushima Medical University
Kaneita, Yoshitaka	Nihon University
Kaneko, Yoshihiro	Akita University
Katanoda, Kota	National Cancer Center
Kato, Noriko	National Institute of Public Health, Japan
Kawamura, Takashi	Kyoto University
Kayaba, Kazunori	Saitama Prefectural University
Kikuchi, Shogo	Aichi Medciacl University
Kita, Yoshikuni	Shiga University of Medical Science
Kiyohara, Yutaka	Kyushu University
Kobashi, Gen	National Institute of Radiological Sciences
Kobayashi, Yasuki	University of Tokyo
Koda, Michiko	Chukyo Women’s University
Kodama, Tomoko	National Institute of Public Health, Japan
Kohyama, Jun	Tokyo Bay Urayasu/Ichikawa Medical Center
Koizumi, Akio	Kyoto University
Kojima, Masayo	Nagoya City University
Kokubo, Yoshihiro	National Cardiovascular Center
Komiya, Hiromi	Fukushima Medical University
Kondo, Naoki	Harvard School of Public Health
Kondo, Takaaki	Nagoya University
Kono, Suminori	Kyushu University
Koriyama, Chihaya	Kagoshima University
Kotani, Kazuhiko	Jichi Medical University
Kuriyama, Shinichi	Tohoku University
Kurokawa, Naoyuki	Tohoku University
Lin, Yingsong	Aichi Medical Uniersity
Liu, Ying	GlaxoSmithKline
Maeda, Kenji	Osaka Medical Center for Health Science and Promotion
Mafune, Kosuke	University of Occupational and Environmental Health, Japan
Marugame, Tomomi	National Cancer Center
Masaki, Motofumi	Nagasaki University
Matsuda, Shinya	University of Occupational and Environmental Health, Japan
Matsuda, Tomohiro	National Cancer Center
Matsuo, Keitaro	Aichi Cancer Center
Metoki, Hirohito	Tohoku University
Minami, Yuko	Tohoku University
Misago, Chizuru	Tsuda College
Miyachi, Motohiko	National Institute of Health and Nutrition
Mizushima, Shunsaku	Yokohama City University
Mochizuki, Yumiko	National Cancer Center
Mori, Mitsuru	Sapporo Medical University
Morioka, Seiji	Tanabe Public Health Center, Wakayama
Morita, Akemi	National Institute of Health and Nutrition
Morita, Manabu	Okayama University
Murakami, Yoshitaka	Shiga University of Medical Science
Nagai, Masaki	Saitama Medical University
Naito, Yoshihiko	Mukogawa Women’s University
Nakagomi, Osamu	Nagasaki University
Nakai, Satoshi	Yokohama National University
Nakamura, Koshi	Kanazawa Medical University
Nakamura, Mieko	Panasonic Electric Works Denro Co., Ltd.
Nakamura, Yasuyuki	Kyoto Women’s University
Nakamura, Yosikazu	Jichi Medical University
Nakata, Yoshio	University of Tsukuba
Nakaya, Naoki	Kamakura Women’s University
Nakayama, Takeo	Kyoto University
Nakayama, Tomohiro	Nihon University
Nanri, Akiko	National Center for Global Health and Medicine
Nishino, Yoshikazu	Miyagi Cancer Center
Nitta, Hiroshi	National Institute for Environmental Studies
Noda, Hiroyuki	Harvard School of Public Health
Nojima, Masanori	Sapporo Medical University
Oba, Shino	National Institute of Public Health, Japan
Ogawa, Hiroshi	Chubu University
Ohira, Tetsuya	Osaka University
Ohkado, Akihiro	Japan Anti-Tuberculosis Association
Ohnishi, Hirofumi	Sapporo Medical University
Ohno, Yuko	Osaka University
Ohsawa, Masaki	Iwate Medical University
Ohshige, Kenji	Yokohama City University
Oida, Yukio	Chukyo University
Ojima, Toshiyuki	Hamamatsu University School of Medicine
Okamoto, Etsuji	National Institute of Public Health, Japan
Okamoto, Naoyuki	Kanagawa Cancer Center
Omori, Takashi	Kyoto University
Ooki, Syuichi	Ishikawa Prefectural Nursing University
Osaki, Yoneatsu	Tottori University
Oshima, Akira	Osaka Medical Center for Cancer and CVD
Ota, Atsuhiko	Fujita Health University School of Medicine
Otani, Tetsuya	National Research Institute for Child Health and Development
Ozasa, Kotaro	Radiation Effects Research Foundation
Sadakane, Atsuko	Jichi Medical University
Saeki, Satoru	University of Occupational and Environmental Health, Japan
Saijo, Yasuaki	Asahikawa Medical University
Saijoh, Kiyofumi	Kanazawa Medical University
Saito, Isao	Ehime University
Saito, Kyoko	Tokyo Metropolitan Geriatric Hospital and Institute of Gerontology
Saito, Tomohiro	National Research Institute for Child Health and Development
Saitoh, Sigeyuki	Sapporo Medical University
Sakamoto, Naoko	National Research Institute for Child Health and Development
Sakata, Kiyomi	Iwate Medical University
Sakauchi, Fumio	Sapporo Medical University
Sakurai, Masaru	Kanazawa Medical University
Sasaki, Hideo	Hiroshima A-Bomb Casualty Council Health Management & Promotion Center
Sasaki, Satoshi	University of Tokyo
Sasazuki, Shizuka	National Cancer Center
Sato, Kyoko	Osaka City University
Satoh, Toshihiko	Kitasato University
Sawada, SS	Tokyo Gas
Sekikawa, Akira	University of Pittsburgh
Shima, Masayuki	Hyogo College of Medicine
Shimazu, Akihito	University of Tokyo
Shimazu, Taichi	National Cancer Center
Shin, Hai-Rim	NCC Korea
Soda, Midori	Radiation Effects Research Foundation
Sokejima, Shigeru	National Institute of Public Health, Japan
Sone, Hirohito	University of Tsukuba
Sonobe, Tomoyoshi	Japanese Red Cross Medical Center
Sozu, Takashi	Kyoto University
Sugimori, Hiroki	Daito Bunka University
Sugimura, Haruhiko	Hamamatsu University School of Medicine
Sugiyama, Hiromi	Radiation Effects Research Foundation
Suka, Machi	Jikei University School of Medicine
Sumi, Ayako	Sapporo Medical University
Suzuki, Kohta	University of Yamanashi
Suzuki, Sadao	Nagoya City University
Tabara, Yasuharu	Ehime University
Taguri, Masataka	Yokohama City University
Takahashi, Hideto	University of Tsukuba
Takahashi, Kunihiko	National Institute of Public Health, Japan
Takakura, Minoru	University of the Ryukyus
Takebayashi, Toru	Keio University
Takeshita, Tatsuya	Wakayama Medical University
Takezaki, Toshiro	Kagoshima University
Tamakoshi, Akiko	Aichi Medical Uniersity
Tamashiro, Hiko	Hokkaido University
Tanabe, Naohito	Niigata University
Tanaka, Hideo	Aichi Cancer Cetner
Tanaka, Junko	Hiroshima University
Tanaka, Keitaro	Saga University
Tanaka, Masahiro	Osaka Medical Center for Cancer and CVD
Tatemichi, Masayuki	Showa University
Teramukai, Satoshi	Kyoto University Hospital
Thomson, Murray	University of Otago
Todoriki, Hidemi	University of the Ryukyus
Tominaga, Suketami	Aichi Cancer Center
Tonda, Tetsuji	Hiroshima University
Toyoshima, Hideaki	Anjo Kosei Hospital
Tsai, Elaine	VA Puget Sound Health Care System
Tsugane, Shoichiro	National Cancer Center
Tsuji, Ichiro	Tohoku University
Tsukuma, Hideaki	Osaka Medical Center for Cancer and CVD
Tsutsumi, Akizumi	University of Occupational and Environmental Health, Japan
Turin, Tanvir	Shiga University of Medical Science
Ueda, Kayo	National Institute for Environmental Studies
Ueshima, Hirotsugu	Shiga University of Medical Science
Wakai, Kenji	Nagoya University
Washio, Masakazu	St. Mary’s College
Watanabe, Makoto	National Cardiovascular Center
Watanabe, Shaw	Life Science Promotion Foundation
Watanabe, Shuichiro	J.F. Oberlin University
Wu, Jiunn-Jong	National Cheng Kung University
Yamagata, Zentaro	University of Yamanashi
Yamaguchi, Naohito	Tokyo Women’s Medical University
Yamaguchi, Takuhiro	University of Tokyo
Yamaji, Taiki	National Cancer Center
Yamamoto, Seiichiro	National Cancer Center
Yamamoto, Tatsuo	Niigata University
Yamanaka, Noboru	Wakayama Medical University
Yamato, Hiroshi	University of Occupational and Environmental Health, Japan
Yamazaki, Shin	Kyoto University
Yanai, Hideki	Fukujuji Hospital
Yasumura, Seiji	Fukushima Medical University
Yatsuya, Hiroshi	Nagoya University
Yokoi, Sana	Chiba Cancer Center
Yokoyama, Eise	Nihon University
Yokoyama, Tetsuji	National Institute of Public Health, Japan
Yoshida, Hiroto	Tokyo Metropolitan Institute of Gerontology
Yoshiike, Nobuo	Aomori University of Health and Welfare
Yoshimura, Kimio	Keio University

